# Simulation CT-based radiomics for prediction of response after neoadjuvant chemo-radiotherapy in patients with locally advanced rectal cancer

**DOI:** 10.1186/s13014-022-02053-y

**Published:** 2022-04-28

**Authors:** Pierluigi Bonomo, Jairo Socarras Fernandez, Daniela Thorwarth, Marta Casati, Lorenzo Livi, Daniel Zips, Cihan Gani

**Affiliations:** 1 Radiation Oncology, Azienda Ospedaliero-Universitaria Careggi, University of Florence, Florence, Italy; 2 Section for Biomedical Physics, Department of Radiation Oncology, Eberhard Karls University Tübingen, Tübingen, Germany; 3 Medical Physics, Azienda Ospedaliero-Universitaria Careggi, University of Florence, Florence, Italy; 4 Department of Radiation Oncology, Medical Faculty and University Hospital, Eberhard Karls University Tübingen, Tübingen, Germany

**Keywords:** Radiomics, Simulation computed tomography, Rectal cancer, Pathologic response, Radiotherapy, Chemotherapy

## Abstract

**Background:**

To report on the discriminative ability of a simulation Computed Tomography (CT)-based radiomics signature for predicting response to treatment in patients undergoing neoadjuvant chemo-radiation for locally advanced adenocarcinoma of the rectum.

**Methods:**

Consecutive patients treated at the Universities of Tübingen (from 1/1/07 to 31/12/10, explorative cohort) and Florence (from 1/1/11 to 31/12/17, external validation cohort) were considered in our dual-institution, retrospective analysis. Long-course neoadjuvant chemo-radiation was performed according to local policy. On simulation CT, the rectal Gross Tumor Volume was manually segmented. A feature selection process was performed yielding mineable data through an in-house developed software (written in Python 3.6). Model selection and hyper-parametrization of the model was performed using a fivefold cross validation approach. The main outcome measure of the study was the rate of pathologic good response, defined as the sum of Tumor regression grade (TRG) 3 and 4 according to Dworak’s classification.

**Results:**

Two-hundred and one patients were included in our analysis, of whom 126 (62.7%) and 75 (37.3%) cases represented the explorative and external validation cohorts, respectively. Patient characteristics were well balanced between the two groups. A similar rate of good response to neoadjuvant treatment was obtained in in both cohorts (46% and 54.7%, respectively; *p* = 0.247). A total of 1150 features were extracted from the planning scans. A 5-metafeature complex consisting of Principal component analysis (PCA)-clusters (whose main components are LHL Grey-Level-Size-Zone: Large Zone Emphasis, Elongation, HHH Intensity Histogram Mean, HLL Run-Length: Run Level Variance and HHH Co-occurence: Cluster Tendency) in combination with 5-nearest neighbour model was the most robust signature. When applied to the explorative cohort, the prediction of good response corresponded to an average Area under the curve (AUC) value of 0.65 ± 0.02. When the model was tested on the external validation cohort, it ensured a similar accuracy, with a slightly lower predictive ability (AUC of 0.63).

**Conclusions:**

Radiomics-based, data-mining from simulation CT scans was shown to be feasible and reproducible in two independent cohorts, yielding fair accuracy in the prediction of response to neoadjuvant chemo-radiation.

**Supplementary Information:**

The online version contains supplementary material available at 10.1186/s13014-022-02053-y.

## Background

Preoperative radiation therapy (RT) is the mainstay of multidisciplinary treatment [1] in locally advanced rectal cancer (LARC). A very high rate (≥ 90%) of sustained loco-regional control is obtained with neoadjuvant concurrent chemo-radiotherapy (CRT) followed by total mesorectal excision (TME) [2]. After surgery, a pathologic complete response (pCR) can be found in 10% to 25% of specimens. A long-term survival benefit [3] was reported for patients for whom no viable tumor cells were detectable following treatment. Thus, pCR is considered a surrogate marker for favorable outcome. In view of its positive prognostic impact but also of the morbidity [4] commonly associated with standard trimodality treatment, a growing interest emerged [5] in pursuing organ preservation strategies. Indirectly, no significant disease-free survival difference was shown between patients with a complete clinical remission on a “watch and wait” policy [6] and those with a proven pCR after TME. Conventional magnetic resonance (MR) imaging plays a pivotal role in staging and prognostication [7] of LARC, however it is characterized by very limited sensitivity [8] in assessing minimal residual disease after CRT. In this perspective, suboptimal diagnostic accuracy was also reported for functional imaging modalities, such as ^18^F-fluorodeoxyglucose (FDG) Positron Emission Tomography/Computed tomography (PET-CT) and diffusion-weighted MR [9]. The inability to predict the response to standard pre-operative treatment is a major limitation in clinical practice. The lack of biomarkers allowing for personalized radiation oncology [10] is an unmet need in LARC. Radiomics is a complex process [11] that consists of the high-throughput extraction of multidimensional features from images, their conversion into mineable data and ensuing support for clinical decision—making. In LARC, radiomics is still in its infancy. Promisingly, the additive value of quantitative data analysis combined with clinical information in identifying disease remission was reported in preliminary studies [12–22] centered on MR imaging. A major advantage of a CT-based radiomics approach is the standardized nature of the imaging information (Hounsfield unit). However, far less information are available in respect to CT-based radiomics in rectal cancer [23–28]. We therefore aimed to evaluate the potential accuracy of a radiation planning CT—based radiomics signature in the prediction of response to neoadjuvant CRT in patients with LARC.

## Methods

### Patients’ and treatment characteristics

We performed a dual-institution, observational, retrospective study. Consecutive patients treated for histologically-confirmed, locally advanced adenocarcinoma of the rectum at the Universities of Tübingen (TU) and Florence (FL) in two subsequent time frames (1/1/07–31/12/10 and 1/1/11–31/12/17 in TU and FL, respectively) were considered for our analysis. In general, staging included Gadolinium-enhanced pelvic MR, iodinated contrast-enhanced CT of the chest and abdomen, and colonoscopy. Clinical stage was defined according to UICC/TNM 7th edition. After multidisciplinary discussion, all patients with UICC stage II/III rectal cancer deemed amenable to undergo a full course of pre-operative radiation-based treatment followed by curatively-intended surgery could be included in our study. No tumor upper distance limit from the anal verge was specified. The primary tumor location was identified based on pelvic MR, in accordance with consensus definition [29]. No upper age limit was defined. Neoadjuvant chemotherapy, unresectable primary tumor, previous RT to the pelvis or previous surgical manipulation of the rectum were exclusion criteria. In addition, patients with unrecognizable rectal Gross Tumor Volume (GTV) on the planning CT or with image artifacts induced by hip prosthesis or rectal stent could not be included. As per local practice, standard of care for neoadjuvant treatment differed between the two centers. In TU, 50.4 Gy were delivered in 28 fractions of 1.8 Gy each (5 fractions per week). Radio-sensitizing chemotherapy consisted of 120-h continuous infusion of 5-fluorouracil during the first and fifth weeks of radiation (daily dose of 1000 mg/m^2^ on days 1 through 5 and 29 through 33, respectively). Selected patients also received deep regional hyperthermia within a clinical trial, which was administered with a Sigma Eye or Sigma-60 applicator up to twice weekly for at least 60 min to a target temperature of 40.5° Celsius, as previously described [30–32]. In FL, a total dose of 45 Gy was delivered with standard fractionation over 5 weeks (25 fractions of 1.8 Gy per day). Chrono-modulated capecitabine was prescribed for the whole RT course at a daily dose of 825 mg/m^2^ BID. After restaging and due time interval (usually 6 to 10 weeks from the end of CRT), surgery was performed. Abdomino-perineal resection or rectal anterior resection (RAR) with TME were the procedures of choice. Pathologic response evaluation was assessed in accordance with Dworak’s tumor regression grade (TRG) [33] in both institutions. Dworak’s 5-point scale was as follows: 0, 1, 2, 3 and 4 scores were indicative of no regression, predominantly tumor with significant fibrosis and/or vasculopathy, predominantly fibrosis with scattered tumor cells, only scattered tumor cells in the space of fibrosis with/without acellular mucin, and no vital tumor cells detectable, respectively. Capecitabine or 5-fluorouracil (plus folinic acid)—based, adjuvant chemotherapy was offered to selected patients in case of unfavorable pathologic findings.

### Imaging analysis and radiomics protocol

**Fig. 1:**
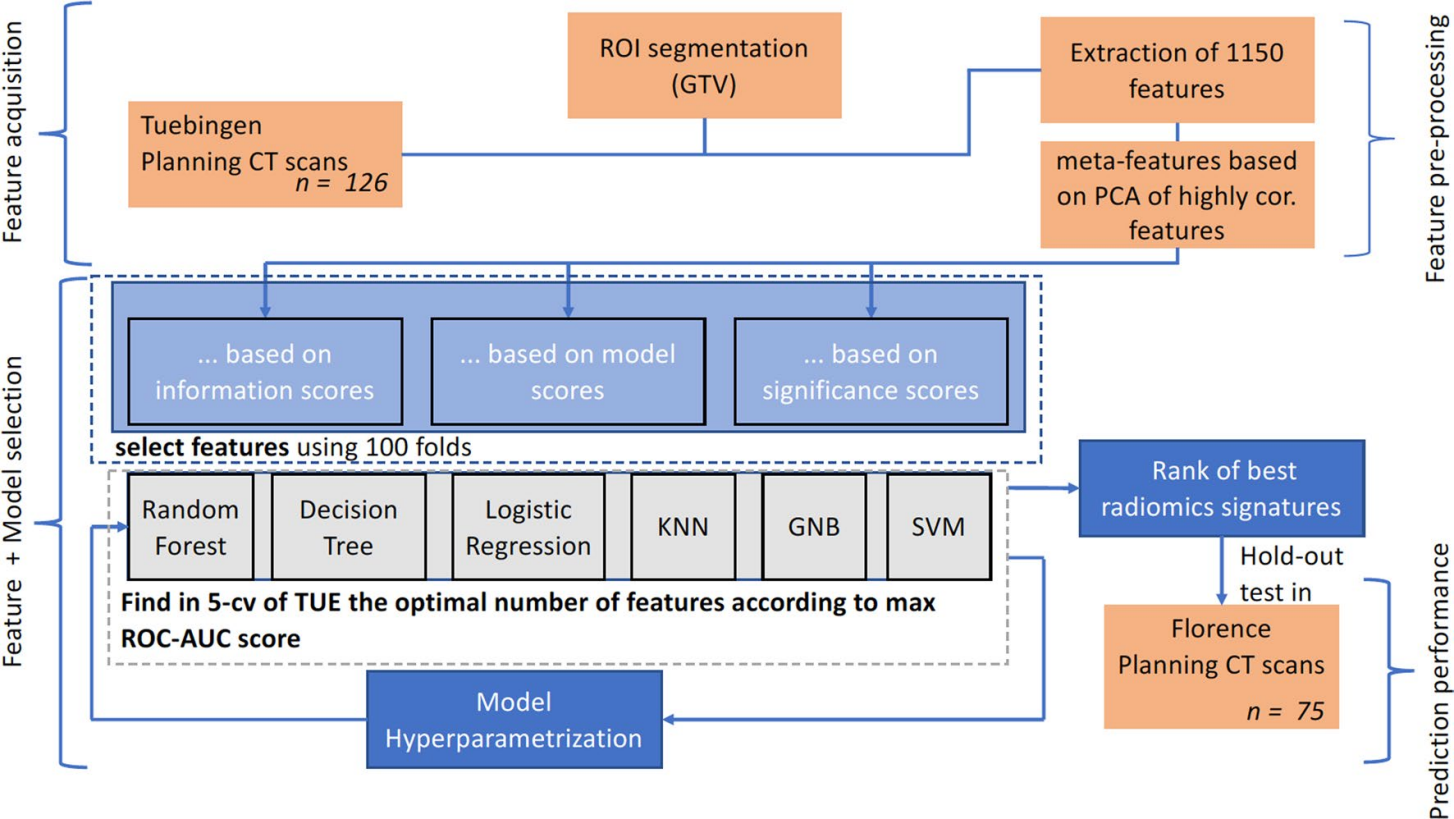
Algorithm workflow for radiomics analysis. PCA, Principal Component Analysis; cor., correlated; GTV, Gross Tumor Volume; CT, Computed Tomography; KNN, k-nearest neighbours; GNB, gaussian naïve-Bayes; SVM, support vector machines; 5-cv, fivefold cross validation; TUE, Tübingen; ROC-AUC score, Receiver Operating Characteristic Curve—Area under the curve

In terms of CT acquisition, treatment planning and delivery, the following procedures were performed, according to local standard of practice. A CT scan (Big Bore, Philips Medical Systems, Cleveland, OH, USA) was acquired at 3 mm slice thickness for planning purpose. The same CT model was used in both institutions. Most patients were immobilized in the prone position with an ankle-holder. In order to displace the small bowel loops from the irradiation field, a belly board device was used. RT was delivered by a linear accelerator (Elekta, Crawley, UK) with standard 3-field box technique or intensity modulated radiotherapy. In terms of delineation, the same procedures were followed in both institutions. The following organs at risk (OAR’s) were contoured: femural heads, bladder, small bowel, penile bulb and anal canal (if not infiltrated). Typically, the clinical target volume (CTV) consisted of the mesorectum and internal iliac, pre-sacral and obturatory lymph nodes. In the definition of CTV, no volume modulation was used. For the purpose of this study all primary tumors were manually segmented by either of two experienced radiation oncologists (CG and PB) in a blinded fashion. For selected cases, such as those with difficult visualization of the GTV, a consensus segmentation between the two physicians was performed. Staging MR T2-weighted sequences was used to aid target definition. Imaging characteristics such as intensity distributions, texture patterns, shape features and wavelets (coiflet 1) kernel based features were extracted from planning CTs of both institutions through volume-averaged and voxelized methods. Features definitions were obtained from the Imaging Biomarker Standardisation Initiative (IBSI) [34]. For the texture features, we used the grey-level co-occurrence (GLCM), grey-level run length (GRLM), neighbourhood grey tone difference (NGTDM), grey-level size zone (GLZSM) and grey-level distance zone (GLDZM) matrix. They were computed in 3 dimensions regardless of differences between in-plane and in-slice voxel dimensions. One level undecimated wavelet features were obtained as follows. Firstly, the original images were filtered using high (H) or low-pass (L) “Coiflet 1” filter in every image (x, y, z) direction. Different filter combinations resulted in 8 filtered images. Subsequently, intensity and texture features were computed for each filtered image [35]. All filtering and feature computations were implemented in-house in Python 3.6. Several of the radiomics features described by the IBSI are highly correlated and therefore redundant. Hence, in the training phase, we clustered correlated features (more than 95% correlation in Pearson correlation coefficient), in order to optimise the feature selection process. To do so, features were first scaled according to the quartile range (interquartile range, IQR), which ranges between the first quartile (25% quantile) and the third quartile (75% quantile). This was performed to avoid strong influence of noisy observations (for instance to imaging artefacts). Then, they were clustered hierarchically according to Pearson correlation coefficient. Finally, every cluster was reduced to one single feature using principal component analysis (PCA) to conserve the maximum possible variance inside the cluster [36]. Moreover, all features with variance lower than 0.3 were excluded from the final feature set. A feature selection process was performed whereby features with low correlation were excluded, highly correlated features were reduced to a single meta-feature and several feature selection algorithms were applied, yielding mineable data through Python 3.6. After feature selection, model hyperparameters such as the number of neighbours for Random Forest (RF) were optimised using grid search (Additional file 1 for full radiomics protocol) and fivefold cross validation. All methods and algorithms were implemented in-house in Python 3.6 using the packages *Pandas*, *Scikit-learn* and *mlxtend* for machine learning. A schematic overview of the algorithmic workflow used in this study is represented in Fig. 1.

### Outcome measures and statistical analysis

After treatment, all patients were followed-up in accordance with international guidelines [37]. The main outcome measure of the study was the rate of pathologic good response (GR), defined as the sum of TRG 3 and 4 according to Dworak’s classification. Baseline demographics, patients’ characteristics and treatment features were summarized using descriptive statistics. Continuous variables (medians) were analyzed with Mann–Whitney test, while Fisher’s exact chi-square test was employed for categorical variables. A *p* value < 0.05 was considered statistically significant. In order to assess the predictive power of the radiomics signature in estimating the development of GR, Receiver Operating Characteristic (ROC) curves were generated to calculate sensitivity, specificity and area under the curve (AUC) values. All statistical analyses were performed by using the statistical software SPSS (SPSS Inc, Chicago, IL, USA) for Windows (version 22). ROC-AUC curves were extracted with support of the Python 3.6 package scikit-learn and matplotlib.

## Results

A total of 222 imaging datasets of patients treated for LARC in TU and FL in the considered time frame were delineated. Ten patients from TU and eleven from FL were excluded due to poor visibility of the primary tumor on CT. Two-hundred and one patients with visible tumors complying with our inclusion criteria were included in our analysis. A total of 126 (62.7%) and 75 (37.3%) of them represented the training and external validation cohorts, repectively. Patients’ characteristics are shown in Table 1. All patients had UICC/TNM stage II or III LARC.

**Table 1:** patient characteristics

Characteristic	No. of patients (%), n = 201	Tübingen cohort (%), n = 126	Florence cohort (%), n = 75	*p* value
**Median age**				
years (IQR)	65 (56–72)	63 (53.7–71.2)	67 (58–74)	0.041
**Sex**				
Male	133 (66.1%)	81 (64.3%)	52 (69.3%)	0.538
Female	68 (33.9%)	45 (35.7%)	23 (30.7%)	
**Staging (**TNM/AJCC 7th ed.)				
cT2N1/N2	11 (5.5%)	7 (5.5%)	4 (5.3%)	0.655
cT3N0	41 (20.4%)	22 (17.5%)	19 (25.3%)	
cT3N1/N2	127 (63.2%)	81 (64.3%)	46 (61.4%)	
cT4anyN	22 (10.9%)	16 (12.7%)	6 (8%)	
**Primary location**				
Low rectum	89 (44.3%)	50 (39.7%)	39 (52%)	0.148
Middle rectum	101 (50.2%)	70 (55.5%)	31 (41.3%)	
High rectum	11 (5.5%)	6 (4.8%)	5 (6.7%)	
**Median time interval (end of RT- surgery)** weeks (IQR)	7 (6–9)	6 (5–7) *	9 (8–11)	< 0.0001

IQR, Interquartile range; RT, Radiotherapy; *: 9 missing values; °: N1 included N1a, N1b, N1c; N2 included N2a and N2b

**Fig. 2:**
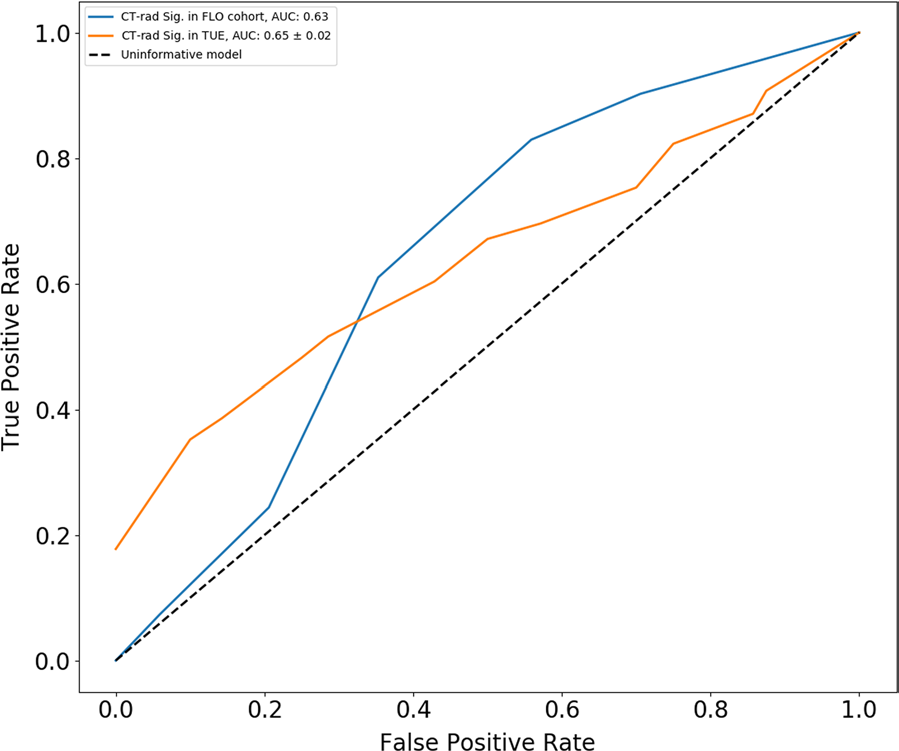
ROC AUC curve for prediction of pathologic good response. CT-Rad Sig: Computed Tomography-Radiomics signature; TUE, Tübingen; FLO, Florence

With the exception of a slighlty younger median age in the German cohort (63 vs 67 years; *p* = 0.042) the remaining patient and tumor features were not significantly different between the two groups. Of note, 78 out of 126 subjects (61.9%) from TU underwent deep regional hyperthermia combined with standard CRT. All included patients underwent surgery at a median interval of 7 weeks (IQR, 6–9) from the end of CRT. A longer median waiting time to intervention was observed in the Italian cohort (9 vs 6 weeks in TU, *p* < 0.0001). In terms of response to treatment, the pCR and GR rates in the overall, TU and FL cohorts were 12.4%, 12.6%, 12%, and 49.2%, 46% and 54.7%, respectively (Table 2). Overall, 201 image datasets from planning CTs were studied and 1150 features were extracted. The most robust 5-metafeature signature consisted of PCA-clusters characterized by LHL Grey-Level-Size-Zone: Large Zone Emphasis (Wavelet-texture family), Elongation (Shape family), HHH Intensity Histogram Mean (wavelet-intensity family), HLL Run-Length: Run Level Variance (wavelet texture family) and HHH Co-occurence: Cluster Tendency (Wavelet—texture family) in combination with 5-nearest neighbour model. When applied to the explorative cohort, the prediction of GR was corresponding to an average AUC value of 0.65 ± 0.02 in 5 cross validation approach. When the model was tested on the external validation cohort, it ensured a similar accuracy, with a slightly lower prediction ability (AUC of 0.63) (Fig. 2).

**Table 2:** pattern of response to neoadjuvant therapy

Characteristic	No. of patients (%), n = 201	Tübingen cohort (%), n = 126	Florence cohort (%), n = 75	*p* value
**TRG**				
0	2 (1%)	2 (1.5%)	0 (0%)	0.459
1	31 (15.5%)	18 (14.4%)	13 (17.3%)	
2	69 (34.3%)	48 (38.1%)	21 (28%)	
3	74 (36.8%)	42 (33.4%)	32 (42.7%)	
4	25 (12.4%)	16 (12.6%)	9 (12%)	
**GR**				
TRG 3 + 4	99 (49.2%)	58 (46%)	41 (54.7%)	0.247

TRG, Tumor Regression Grade; GR, Good Response

## Discussion

To the best of our knowledge, with just over 200 patients our study represents the largest planning CT-based radiomics investigation involving two independent institutions for the prediction of pathologic response after neoadjuvant treatment in rectal cancer. Taking into account the well-known inability of standard clinical parameters in anticipating the response of LARC to neoadjuvant treatment [5], retaining a similar predictive accuracy when shifting from internal to external validation underlines the potential generalizability of our hypothesis-generating data in the clinic. With radiomics, it is hypothesized that selected quantitative features might represent imaging biomarkers [38, 39] able to meaningfully provide information on the prognosis of the disease or the prediction of response to therapies, on top of genomic and metabolic factors. By focusing on simulation CT, we sought to address whether an integral imaging component of radiation workflow may be informative of patient response to treatment and be associated with a favorable phenotype. MR imaging has undisputed relevance in the management of rectal cancer, representing the gold standard modality [40] in this disease. In a single-center, retrospective experience on 48 patients, Nie et al. [12] were the first to show that an artificial neural network approach applied to multi-parametric MR images was able to significantly improve their predictive value of response to CRT, in comparison with conventional parameters. In a more recent multi-center study on 226 cases, Dinapoli and colleagues [14] demonstrated that a histogram-based radiomics signature could be associated with the development of pCR and that the model was equally informative and reproducible in 3 independent cohorts, independently from the type of MR scanner used. Taking all data on MR-radiomics together [12–22], albeit promising, the large heterogeneity and complexity observed in terms of imaging standardization [41] restrain from direct clinical application in LARC.

In colo-rectal cancer, CT-based radiomics modelling was thus far explored in regards to preoperative stage discrimination [42], identification of pathologic lymph nodes [43], and grading differentiation [44]. To the best of our knowledge, only few groups [23–28] focused on the potential predictive value in respect to pathologic response to treatment. In a single-center, retrospective analysis [23] on 95 patients who received neoadjuvant CRT, homogeneous texture features (≤ 6.7 for entropy, ≥ 0.0118 for uniformity, and ≤ 28.06 for standard deviation) extracted from diagnostic, contrast-enhanced CT scans correlated with better disease-free survival. Vandendorpe et al. [24] performed a similar explorative analysis on baseline staging CTs of 121 patients, split between a training and validation cohorts (79 and 42 subjects, respectively). Of note, 84.3% of the whole sample was selected from a single institution. Texture analysis was based on a single slice of portal-phase images where the ROI was delineated by only one radiologist. The model showed good discriminative ability to predict a downstaging response in the training cohort (AUC of 0.90, 95% CI 0.83–0.97) which however did not hold in the test data set (AUC of 0.70, 95% CI 0.48–0.92). The use of simulation CT for radiomics modelling was previously described in a single experience. Bibault and colleagues [25] performed a high-dimensional quantitative analysis by creating a deep neural network (DNN) based on a combination of clinical information (primary tumor stage) and 28 features. The latter were extrapolated from planning CT scans of 95 patients with LARC treated in 3 different institutions. Through their innovative approach, the authors showed that the tested DNN yielded an 80% accuracy in the prediction of pCR, with a mean AUC value of 0.72 (95% CI 0.65–0.87). Other than powerful calculation algorithms, the methodological quality of investigations on radiomics must be considered of utmost importance, particularly when—as in ours and many previously published experiences—no “ground truth” correspondence between imaging and biology is available. For this purpose, Lambin and colleagues [45] proposed a composite metrics, the so called “quality radiomics score” (QRS), to evaluate the overall quality of a radiomic worflow. Secondly only to a prospective study design registered in a clinical trial database, the presence of validation in at least two distinct datasets from independent institutions is considered the single most important factor, with a score of 4 out of a total of 36 points. When taking into account the TRIPOD statement [46] as a tool to assess the value of predictive models, our work should be regarded as a type 3 study, ranking therefore among the most reliable investigations. In the context of quantitative imaging analysis, the use of a highly standardized imaging modality such as simulation CT is also something worth highlighting. In view of its inherent reproducibility, a potential cross-validation among radiation oncology centers from different countries may not represent a critical issue as in the case of more complex diagnostic procedures, such as MR or FDG PET-CT. In this perspective, planning CT scans could well be viewed as truly “theragnostic” [47, 48] images characterized by reduced inter-operator variability, widespread accessibility, and cost-effectiveness. Pending extensive validation, the versatility of CT-based radiomics could pave the way for longitudinal analysis [49], integration of 3D dose distribution [50–52] (or dosiomics) and more complex applications in the frame of hybrid [53, 54] machines. In analogy with previous published experiences [23, 25], the presence of radiomics features portending lower heterogeneity in addition to an elongated shape in our 5-metafeature combination may be indicative of better response to neoadjuvant treatment. In view of very similar results in both TU and FL cohorts, pointing towards its reproducibility, the fair predictive accuracy of GR of the identified radiomics signature (AUC > 0.6 < 0.7) could depend on several reasons. In our retrospective study, the presence of unrecognized confounding variables can’t be overlooked. In particular, factors such as treatment heterogeneity, waiting time to surgery [55], different RT total dose and type of systemic agents may have well influenced the pattern of response after treatment. However, patient features were not significantly different between the two cohorts, with the exception of a slightly younger median age in the German group (63 vs. 67 years; *p* = 0.042). A longer median waiting time to intervention was observed in the Italian cohort (9 vs. 6 weeks in Tuebingen, *p* < 0.0001). The shorter time interval in the Tuebingen cohort may be due to the fact that patients were treated in an older period compared with those in the Florence one, mirroring a different attitude towards what has become part of routine practice more recently. However, still today the interplay between pathologic response after neoadjuvant treatment and waiting time to surgery is among the most controversial research topics in rectal cancer, without definitive answers [56, 57]. Both median time intervals in our study groups fell within the acceptable standard of care, and no significant difference was reported in terms of pCR and GR between the two cohorts. Deep regional hyperthermia is a well known strategy with potential radiosensitizing effect, with relatively limited applications in the clinic in experienced centers. Albeit promising, relatively limited prospective data are available in rectal cancer [5]. A recently published single-arm, prospective phase 2 trial [32] showed optimal outcome and compliance by adding hyperthermia to concomitant CRT in patients with LARC, with an overall pCR rate of 14%, very similar to what was found in our work. Although we can’t rule out that the use of hyperthermia in 61.9% of patients from the Tuebingen cohort may have contributed to a better response, a marked unbalance on our results is unlikely, in our opinion. Overall, no clinical variables emerged in our model as significant, so that no stratification for T and N stage was performed. Further limitations have to be acknowledged in the interpretation of our results. First, the relatively small sample size and the reported pCR rate at the lower end of the expected range limit the strength of our findings. In view of the small proportion of patients with pCR in our study, we decided to cluster patients with TRG 3 and 4. However, since TRG 3 is indicative of only few scattered tumor cells left in the specimen, the biological meaning of it can be very similar to TRG 4. In the first ever published experience on radiomics in rectal cancer [12] a similar approach was applied to discriminate good responders from the rest. Second, the intrinsically low contrast resolution of native CTs and the overall image quality may have hampered the accuracy of GTV segmentation in some challenging cases, in spite of our attempt to solve discrepancies by consensus between the two radiation oncologists involved in target delineation. In this perspective, tools for semi-automatic segmentation may provide benefit, particularly for the definition of cranio-caudal extension [58]. Third, tumor volume was not predictive of GR in our study and thus excluded from the final model; however, it cannot be overlooked that our radiomics signature could be just a surrogate of tumor volume [59] and as such associated with pathologic response, as already shown in other investigations with similar discriminative power. Fourth, when dealing with the “classical” rule of thumb introduced by Hosmer and Lemeshow [60] on how to score the discriminative ability of a ROC curve, a minimum AUC score of 0.7 defines the lower end of what can provide an acceptable discrimination. However, in a hypothesis-generating study such as ours even an AUC value > 0.6 < 0.7 may still be deemed fair and worthy of further investigation. For instance, to name one of the most studied examples in radiation oncology involving quantitative imaging, when applying radiomics analysis to NTCP models for the prediction of radiation pneumonitis, AUC values between 0.6 and 0.7 have been regarded as promising [61, 62]. Albeit the disciminative power by itself in our stud was not very high, we believe that our CT-based radiomics model is strenghtened by the independent, dual-institution validation approach we performed and very similar performance we obtained in both cohorts, which may represent a benchmark for future investigations on simulation CT in LARC. Ultimately, recognizing the central role of MR imaging in rectal cancer and the fact that by itself CT imaging may not be ideal in terms of GTV definition, we believe that in terms of radiomics perspective, the use of simulation CT for radiomics purposes may be of extreme interest, given the fact that it is essentially imaging acquired in the treatment position, in contrast to diagnostic MR. Our study lends support to CT-based radiomics in LARC as a hypothesis-generating approach, adding value to the very limited available evidence in this topic, pending further external validation in a larger cohort of patients.

## Conclusions

In our hypothesis-generating study, a process of data-mining from pre-treatment simulation CT scans was shown to be feasible and reproducible in two independent cohorts. The potential predictive ability of a CT-based signature in both datasets in identifying patients with pathologic GR after surgery warrants further investigations.

## Supplementary Information

**Additional file 1**. Full radiomics protocol.

## Data Availability

The present data is summarized in this paper (METHODS). The complete dataset can be retrieved from the authors upon formal request from interested readers.

## Abbreviations

CT: Computed tomography
PCA: Principal component analysis
AUC: Area under the curve
RT: Radiation therapy
LARC: Locally advanced rectal cancer
CRT: Chemo-radiotherapy
TME: Total mesorectal excision
pCR: Pathologic complete response
MR: Magnetic resonance
FDG: 18F-fluorodeoxyglucose
PET-CT: Positron Emission Tomography/Computed tomography
TU: Tuebingen
FL: Florence
UICC: Union Internationale Contre Le Cancer
GTV: Gross tumor volume
RAR: Rectal anterior resection
TRG: Tumor regression grade
OH: Ohio
UK: United Kingdom
USA: United States of America
OAR’s: Organs at risk
CTV: Clinical target volume
CG: Cihan Gani
PB: Pierluigi Bonomo
IBSI: Imaging Biomarker Standardisation Initiative
GLCM: Grey-level co-occurrence
GRLM: Grey-level run length
NGTDM: Neighbourhood grey tone difference
GLZSM: Grey-level size zone
GLDZM: Grey-level distance zone
H: High
L: Low
IQR: Interquartile range
RF: Random forest
GR: Good response
ROC: Receiver operating characteristic
AUC: Area under the curve
DNN: Deep neural network
QRS: Quality radiomics score
TRIPOD: Transparent reporting of a multivariable prediction model for individual prognosis or diagnosis
3D: 3 Dimensional
