# Supplementary material for: Simulation CT-based radiomics for prediction of response after neoadjuvant chemo-radiotherapy in patients with locally advanced rectal cancer
Source: Radiat Oncol. 2022 Apr 28;17:84. doi: 10.1186/s13014-022-02053-y (PMC9052564; doi:10.1186/s13014-022-02053-y)
Supplement: Supplementary file 1 — Additional file 1. Full radiomics protocol. [file 13014_2022_2053_MOESM1_ESM.docx]

**Title:** Simulation CT - based radiomics for prediction of response after neoadjuvant chemo-radiotherapy in patients with locally advanced rectal cancer

**Supplementary material: Radiomics protocol**

*Imaging pre-processing and feature extraction*

For the radiomics analysis, CT images were used without voxel resizing, in order to avoid inclusion of artificial information that might cause noise at the moment of feature calculations. Only soft tissue voxels with values between -250 and 120 HU were considered in order to exclude air, and other non-tissue elements. 64 bins were used to group voxel values for texture feature calculations.

Features definitions were obtained from the Imaging Biomarker Standardisation Initiative (IBSI) (1). For the texture features, we used the grey-level co-occurrence (GLCM), grey-level run length (GRLM), neighbourhood grey tone difference (NGTDM), grey-level size zone (GLZSM) and grey-level distance zone (GLDZM) matrix. They were computed in 3 dimensions regardless of differences between in-plane and in-slice voxel dimensions. One level undecimated wavelet features were obtained as follows. Firstly, the original images were filtered using high (H) or low-pass (L) “Coiflet 1” filter in every image (x, y, z) direction. Different filter combinations resulted in 8 filtered images.  Subsequently, intensity and texture features were computed for each filtered image (2). In summary, we extracted 1150 radiomics features from GTV regions contoured in the planning CT scans. All filtering and feature computations were implemented in-house in Python 3.6.

*Feature pre-processing*

Several of the radiomics features described by the IBSI are highly correlated and therefore redundant. Hence, in the training phase, we clustered correlated features (more than 95% correlation in Pearson correlation coefficient), in order to optimise the feature selection process. To do so, features were first scaled according to the quartile range (interquartile range, IQR), which ranges between the first quartile (25% quantile) and the third quartile (75% quantile). This was performed to avoid strong influence of noisy observations (for instance to imaging artefacts). Then, they were clustered hierarchically according to Pearson correlation coefficient. Finally, every cluster was reduced to one single feature using principal component analysis (PCA) to conserve the maximum possible variance inside the cluster (3). Moreover, all features with variance lower than 0.3 were excluded from the final feature set.

A four-step feature selection method was implemented as follows:

1. Step 1: the training cohort was randomly subsampled with replacement in a balanced fashion so that each subsample contains the same number of positive and negative class. This was repeated 100 times, thus creating a set of 100 subsamples.
2. Step 2: within each subsample, variable importance was determined using:

- correlation measures (Pearson (4), Kendall (5), Spearman (6),
- mutual information (mutual information maximisation (7),
- univariate significance test scores (Fischer, ᵡ^2^) (8),
- multivariate forward selection using classification models (decision trees (DT), k-nearest neighbours (KNN), logistic regression (LogR), random forest (RF), gaussian naïve-Bayes (GNB), support vector machines (SVM) based on the model Receiver Operating Characteristic Curve -  Area under the curve (ROC-AUC) score (9).

For all methods above, up to twenty most important features were kept, and the remaining features were discarded. These feature subsets were then aggregated across the different methods to form a final subset of the five most commonly occurring features for each subsample to rank the most stable ones.

1. Step 3: the features in the final subset of each of the 100 subsamples were then aggregated and heuristically ranked using the following scoring:

$RS=\left( n_{A}/100 \right)\left( \mu_{r} \left( \sigma_{r}+1 \right) \right)^{-1}$ .

The scoring favours the number of appearances (na) of a feature in the 100 subsets, and penalises its mean rank $\mu_{r}$ together with the standard deviation of its rank $\sigma_{r}$ in the subsets. The five most highly ranked features were subsequently selected.

1. Step 4: We determined the signature for each of the classifiers using a sequential forward feature selection method (10). For this purpose, we performed 5-fold cross-validation using the TU data set. For each classifier, the set of features that produced the model with the highest average AUC on the validation folds was used as a signature.

After feature selection, model hyperparameters such as the number of neighbours for RF were optimised using grid search (table 1) and 5-fold cross validation. All methods and algorithms were implemented in-house in Python 3.6 using the packages *Pandas*, *Scikit-learn* and *mlxtend* for machine learning.

| **Model** | **Hyperparameter** | **Hyperparameter space** |
| --- | --- | --- |
| Logistic Regression | Penalty | ‘L1’, ‘L2’ |
|  | C | 10^-3^, 10^-2^, 10^-1^, 1, 10, 10^2^, 10^3^, 10^4^ |
|  | Class Weight | {0: 1, 1:2}, {0: 1, 1:3}, {0: 1, 1:4}, {0: 1, 1:5} |
| Nearest Neighbours | Number of Estimators | 3, 5, 7, 9, 11, 13, 15, 17, 19, 21, 23, 25 |
|  | Weights | Uniform, Distance |
| Support Vector Machine | C | 10^-3^, 10^-2^, 10^-1^, 1, 10, 10^2^, 10^3^, 10^4^ |
|  | Kernel | RBF, Polynomial, Sigmoid |
| Random Forest | Criterion | Gini, Entropy |
|  | Number of Estimators | 3, 5, 7, 9, 11, 13, 15, 17, 19, 21, 23, 25 |
|  | Class Weight | {0: 1, 1:2}, {0: 1, 1:3}, {0: 1, 1:4}, {0: 1, 1:5} |
|  | Max Depth | 8, 9, 10, None |
| Gaussian Naïve Bayes | None | None |
| Decision Tree | Criterion | Gini, Entropy |
|  | Class Weight | {0: 1, 1:2}, {0: 1, 1:3}, {0: 1, 1:4}, {0: 1, 1:5} |
|  | Max Depth | 8, 9, 10, None |

Table 1. Description of the used hyperparameter space for each machine learning model. Hyperparameter class weight is a python dictionary which indicates penalization for wrong guessing one of the label class in the model.

*Meaning of the relevant features*

- Elongation (shape family): is a measure of the squared root of the ratio between minor and the largest axis length. Meaning that if a tumour could be enclosed by an ellipsoid, this is a measured of the disproportion of the tumour or in other words how elongated is the tumour.
- LHL Grey-Level-Size-Zone: Large Zone Emphasis (Wavelet-texture family): first, grey level size zone Large Zone emphasis, counts how many large zones (voxels-values that in a neighbourhood are similar to each other) exists in a tumour. An LHL Grey-Level-Size-Zone: Large Zone Emphasis is then a measurement of the LHL Grey-Level-Size-Zone: Large Zone Emphasis in a X low pass filtered (L), Y high pass filtered (H) and Z low pass filtered (L) image volume taken from the original image volume.
- HHH Intensity Histogram Mean (wavelet-intensity family): Measures the mean of the intensity in a X high filtered, Y high filtered, Z high filtered image volume taken from the original image volume
- HLL Run-Length: Run Level Variance (wavelet texture family): measure the variance of counts of voxels with similar voxel values in a determined direction (x, y, z, and combinations) in a image volume which a X high pass filter, Y low pass filter and Z low pass filter was applied.
- HHH Co-occurrence: Cluster Tendency (Wavelet - texture family): is the measurement of the tendency in a neighbourhood of 9 voxels to have similar values in a X high pass filter, Y high pass filter, Z high pass filter, the larger the value the most homogeneous is the filtered image (more similar high repetitive patterns)

In summary: the relevant features suggest that positive class can be predicted from a combination of measurement of the elongation of the tumour in combination of certain homogeneity in certain regions of the image.

**References**

1. Zwanenburg A, Vallières M, Abdalah MA, et al. The Image Biomarker Standardization Initiative: Standardized Quantitative Radiomics for High-Throughput Image-based Phenotyping. Radiology 2020;295(2):328-338. doi: 10.1148/radiol.2020191145.
2. Gonzalez RC, Woods RE, Eddins SL. Digital Image Processing Using MATLAB®. Gatesmark Publishing; 2009.
3. Walker M, Kublin JG, Zunt JR. Fast R functions for robust correlations and hierarchical clustering. J Stat Softw. 2009;42:115–25.
4. Benesty J, Chen J, Huang Y, Cohen I. Pearson correlation coefficient. Noise Reduct. speech Process., Springer; 2009, p. 1–4
5. Abdi H. The Kendall rank correlation coefficient. Encycl Meas Stat Sage, Thousand Oaks, CA 2007:508–10.
6. Spearman Rank Correlation Coefficient. Concise Encycl. Stat., New York, NY: Springer New York; 2008, p. 502–5. doi:10.1007/978-0-387-32833-1_379
7. Peng H, Long F, Ding C. Feature selection based on mutual information: criteria of max-dependency, max-relevance, and min-redundancy. IEEE Trans Pattern Anal Mach Intell 2005;1226–38
8. Huberty CJ, Morris JD. Multivariate analysis versus multiple univariate analyses. Psychol Bull 1989;105:302.
9. Bishop CM. Pattern recognition and machine learning. Springer; 2006
10. Pudil P, Novovičová J, Kittler J. Floating search methods in feature selection. Pattern Recognit Lett. 1994;15:1119–25.
